# Effect of β-mannanase supplementation in low-energy and low-protein diets on performance, intestinal morphology, and fatty liver incidence in laying hens

**DOI:** 10.5713/ab.25.0214

**Published:** 2025-06-10

**Authors:** Kang Hyeon Kim, Eun Cheol Lee, Charline Mugeniwayesu, Tae Hyun An, Dong Yong Kil

**Affiliations:** 1 Department of Animal Science and Technology, Chung-Ang University, Anseong, Korea

**Keywords:** β-mannanase, Fatty Liver, High-mannan Ingredient, Laying Hen, Low-energy and Low-protein Diet, Performance

## Abstract

**Objective:**

This study aimed to investigate the effect of dietary β-mannanase supplementation in low-energy and low-protein diets containing palm kernel meal and copra meal on productive performance, egg quality, intestinal morphology, and fatty liver incidence in laying hens.

**Methods:**

A total of four hundred 26-wk-old Hy-Line Brown laying hens were allotted to 1 of 5 dietary treatments with 8 replicates. The positive control (PC) diet was prepared with corn and soybean meal, whereas the negative control (NC) diet was formulated with decreased AME_n_ by 100 kcal/kg and CP by 0.85% than PC diets. High-mannan NC diet was also prepared by inclusion of 2.5% palm kernel meal and 2.5% copra meal, which was designed to contain energy and nutrient concentrations equal to those in the NC diet. Finally, dietary β-mannanase was supplemented to the high-mannan NC diet at the levels of 0.05% and 0.10%.

**Results:**

Most productive performance and egg quality were not affected by dietary treatments. For jejunal morphology, villus height:crypt depth (VH:CD) ratio for hens fed PC diets or NC diets was greater (p<0.05) than those fed high-mannan NC diets, but supplementation of β-mannanase in high-mannan NC diets did not affect VH:CD ratio in hens. Hens fed NC diets had a greater (p<0.05) subjective color score in the liver than those fed PC diets or high-mannan NC diets supplemented with 0.05% and 0.10% β-mannanase.

**Conclusion:**

Most productive performance and egg quality in laying hens were not affected by reduction in dietary energy and protein levels, inclusion of high-mannan ingredients, and dietary β-mannanase supplementation. No considerable benefits of dietary β-mannanase supplementation in low-energy and low-protein diets containing high-mannan ingredients on productive performance and health were observed in laying hens.

## INTRODUCTION

The recent increase in the cost of the major feed ingredients, such as corn and soybean meal (SBM), pushes poultry nutritionists to increase the use of alternative ingredients and byproducts, such as palm kernel meal (PKM) and copra meal (CM) in poultry diets. However, these ingredients contain high amounts of non-starch polysaccharides (NSPs) that can hardly be digested and utilized in the gastrointestinal tract (GIT) of poultry [1]. In particular, PKM and CM contain high amounts of β-mannan as soluble NSPs, which have been considered the antinutritional factor impairing nutrient digestion and utilization by increasing digesta viscosity in the GIT of poultry [2].

Dietary supplementation of β-mannanase has been widely adopted in poultry diets, especially for diets formulated with high-mannan ingredients, because dietary β-mannanase can break down β-mannan in the GIT, thereby mitigating the negative impact of β-mannan on poultry performance and health [3]. Moreover, several previous studies have reported that the use of β-mannanase in poultry diets is a cost-effective strategy because dietary β-mannanase supplementation maintained a similar productive performance despite decreased energy concentrations in diets [4,5]. The possible reason has been involved in improved nutrient and energy utilization by lowering digesta viscosity in the GIT [3,6]. Furthermore, the recent issues of nitrogen excretion in the soil and greenhouse gas emission from nitrous oxide (N_2_O) lead poultry nutritionists to formulate diets with reduced protein levels and increased amino acid (AA) supplementation [7,8]. Therefore, to realize the benefits of this low-protein diet, the appropriate use of NSP-degrading enzymes (NSPase) is essential because NSPase has been reported to improve nitrogen utilization in addition to other nutrient utilization [9,10]. However, the information regarding the effect of dietary β-mannanase supplementation in low-protein diets on productive performance, health, and nutrient utilization in poultry is limited. Consequently, the recent issues of increasing feed costs and environmental problems facilitate the use of low-energy and low-protein diets in the poultry industry. However, there have been few studies investigating the effect of dietary β-mannanase supplementation in low-energy and low-protein diets particularly formulated with high-mannan ingredients in poultry.

Therefore, this study aimed to investigate the effect of dietary β-mannanase supplementation in low-energy and low-protein diets formulated with PKM and CM on productive performance, egg quality, intestinal morphology, and fatty liver incidence in laying hens.

## MATERIALS AND METHODS

### Animals, diets, and experimental design

A total of four hundred 26-wk-old Hy-Line Brown laying hens with an average body weight (BW) of 2.04 kg were randomly assigned to 1 of 5 dietary treatments with 8 replicates per treatment. Each replicate had 10 cages with 1 hen per cage (24×36×39 cm). All hens were fed a commercial layer diet before the beginning of this study. The positive control (PC) diet was prepared mainly with corn, SBM, and corn gluten meal. The energy and nutrient concentrations in the PC diet were formulated to meet or exceed their recommendations for the economical performance of laying hens in the Hy-Line Brown nutritional guideline [11]. The calculated concentrations of AME_n_ and CP in the PC diet were 2,950 kcal/kg and 17.0%, respectively. The negative control (NC) diet with low-energy and low-protein levels was formulated to contain 2,850 kcal/kg AME_n_ and 16.15% CP. However, calculated concentrations of other nutrients, including digestible AAs, total Ca, and available P, were equalized between PC and NC diets. One additional diet containing high amounts of β-mannan were prepared with inclusion of 2.5% PKM and 2.5% CM with a partial replacement of corn and SBM (High-mannan NC diet), which was designed to contain energy and nutrient concentrations equal to those in the NC diet. Finally, dietary β-mannanase was supplemented to the high-mannan NC diet at the levels of 0.05% and 0.10% (CTCZYME; declared activity of 800,000 U/kg; CTCBIO) in replace of celite. The nutritional compositions of PKM and CM used in this study are presented in Table 1, whereas those in treatment diets, including PC, NC, and high-mannan NC diet, are presented in Table 2. All diets were prepared in a mash form. Treatment diets and water were provided *ad libitum* for 8 wks of the feeding trial. The average temperature and relative humidity were maintained at 24.4±4.8°C and 62.1±19.42% by a periodic ventilation during the entire experiment. A lighting program was weekly adjusted based on the age of laying hens, following the Hy-Line Brown management guideline [11].

### Productive performance and egg quality

Productive performance, including hen-day egg production, egg weight, egg mass, and the rate of broken and shell-less egg production was measured daily during 8 wks of the feeding trial. The feed intake (FI) and feed conversion ratio (FCR) were determined at the end of the experiment. Egg mass was estimated by multiplying egg weight by hen-day egg production. Egg quality, including eggshell color, egg yolk color, eggshell strength, eggshell thickness, and Haugh unit, was analyzed using 10 eggs per replicate, with 5 eggs collected per d at the end of experiment. Eggshell color was determined using the eggshell color fan (Samyangsa) with scales from 1 to 15. Eggshell strength, eggshell thickness, egg yolk color, and Haugh unit were determined by a digital egg tester (DET-6000; Nabel). The detailed procedures for egg quality measurements were described in our previous study [12].

### Sample collection and analysis

The sample of PKM and CM were analyzed for dry matter (DM; AOAC Method 934.01 [13]), nitrogen (N; AOAC Method 984.13; [13]), acid-hydrolyzed ether extract (AEE; AOAC Method 996.01 [13]), and gross energy using bomb calorimeter (Model 6400; Parr Instruments). The neutral detergent fiber and acid detergent fiber concentrations in both PKM and CM were analyzed using an ANKOM 200 Fiber Analyzer (ANKOM Technology) based on the modified method described by Van Soest et al [14]. The concentrations of AA in the PKM and CM were determined using L8900 AA analyzer (Hitachi) according to the methods as described by AOAC (Method 982.30 [13]). The concentrations of calcium (Ca) and phosphorus (P) in the PKM and CM were also analyzed using an inductively coupled plasma spectrometer (Opima 5300 DV; Perkin Elmer), as demonstrated by AOAC (Method 984.27 [13]) with minor modifications [15].

At the cocnlusion of the experiment, all hens were fasted overnight, and the BW of laying hens was measured individually. One hen with a BW close to the average BW of each replicate was selected and euthanized by CO_2_ asphyxiation. The samples for the blood, liver, and jejunum were collected immediately. One more hen with average BW was also selected to measure cutaneous basophil hypersensitivity (CBH) as the cell-mediated immune response. Toe skin thickness was calculated using a micrometer (Thermo Fisher Scientific) at 6, 12, and 24 h after PHA-P injection. The value for the CBH responses was calculated as reported by Corrier and DeLoach [16].

Blood samples were immediately obtained from a heart puncture by using a 6.0-mL BD vacutainer serum tube (BD). The serum samples were sent to HLB BioStep to measure the concentrations of aspartate aminotransferase (AST), alanine aminotransferase (ALT), uric acid, and creatinine. In addition, blood heterophil to lymphocyte ratio (H:L ratio) was assessed as a stress indicator. Blood H:L ratio was determined using the method described by Lentfer et al [17] with a minor modification.

Jejunal morphology was measured by the method of our previous study [15]. Briefly, jejunum samples were flushed and stored at 10% buffered formalin. A 5-mm section of the sample was placed onto a slide glass, stained with hematoxylin-eosin, and measured under a light microscope. Villus height (VH), crypt depth (CD), and VH to CD ratio (VH:CD) were measured with 20 measurements per replicate. The detailed method was described in the previous study [18]. This analysis was performed at the BT research facility center, Chung-Ang University.

Fatty liver incidence was measured based on the method of our previous study [12]. The liver was observed to measure the subjective fatty liver score on a scale of 1 to 5 (1 = dark red; 5 = yellowish red). The liver hemorrhagic score was also analyzed by using a scale from 0 to 5 (0 = normal liver; 5 = large and massive hemorrhages). Three observers conducted the liver scoring in the blind test. In addition, the Commission Internationale de l’Eclairage (CIE) color scales for the lightness (L*), redness (a*), and yellowness (b*) in the liver were measured using a calorimeter (Model CR-10; Konica Minolta Optics). Finally, tissue samples from the middle part of the liver were used to analyze AEE concentrations in the liver (AOAC method 996.01 [13]).

### Statistical analysis

All data were analyzed by one-way ANOVA as a completely randomized design using the PROC MIXED procedure of SAS (SAS Institute). Each replicate was used as an experimental unit for all analyses. The outlier data were checked by the UNIVARIATE procedure of SAS. The LSMEANS procedure was used to calculate the treatment means and the PDIFF option was used to separate the treatment means. A probability of p<0.05 was considered significant and 0.05≤p≤0.10 was considered a tendency.

## RESULTS AND DISCUSSION

### Productive performance and egg quality

Productive performance in laying hens, including hen-day egg production, egg weight, egg mass, FCR, and broken and shell-less egg production rate, was not affected by dietary treatments (Table 3). However, hens fed PC diets had less FI (p<0.05) than those fed NC diets or high-mannan NC diets. Moreover, feeding high-mannan NC diets with 0.10% β-mannanase supplementation showed similar FI to feeding PC diets. However, no such an effect on FI was observed by dietary supplementation of 0.05% β-mannanase in high-mannan NC diets.

Increased FI by feeding low-energy and low-protein diets as observed in this study indicates that laying hens show a compensatory increase in FI for the purpose of meeting their energy and nutrient requirements. Similar results have been observed in previous layer studies [19,20]. Interestingly, dietary supplementation of 0.10% β-mannanase in high-mannan NC diets resulted in a similar FI to PC diets. This result may be related to improved energy and nutrient utilization in poultry by dietary β-mannanase [19,21]. However, reduction in dietary energy and protein levels, the use of high-mannan ingredient, and dietary β-mannanase supplementation had little effects on other productive performance in this study. This result is contradictory to the previous findings that dietary β-mannanase supplementation improved productive performance in laying hens [9,19,22]. The reason for this contrasting result is not clear; however, it may be related to the variation in the age of hens, diet compositions, β-mannan contents, and experimental conditions among studies.

Egg quality, including eggshell color, eggshell strength, eggshell thickness, and Haugh unit, was not influenced by dietary treatments (Table 4). However, hens fed NC diets had the least (p<0.05) egg yolk color among treatments, whereas those fed high-mannan NC diets with 0.10% β-mannanase had lower (p<0.05) egg yolk color than those fed other high-mannan treatment diets.

As observed in productive performance, most egg quality was not affected by reduction in dietary energy and protein levels, the use of high-mannan ingredient, and dietary β-mannanase supplementation. The major reason for this observation may be due to the fact that laying hens used in this study were placed in the early and peaking production stage and egg quality in this production stage is generally very high during the overall production cycle. Therefore, it is likely that dietary treatments had little impacts on egg quality in this study. However, egg yolk color was the least for hens fed NC diets among treatments. The reason for this observation is that PC diets and high-mannan NC diets were formulated with high inclusion of corn gluten meal that contains high amounts of carotenoids as egg yolk pigments [23].

### Jejunal morpholgy

No effects of dietary treatments on jejunal VH and CD in laying hens were observed (Table 5). However, jejunal VH:CD ratio for hens fed PC diets or NC diets was greater (p<0.05) than those fed high-mannan NC diets, but dietary supplementation of 0.05% or 0.10% β-mannanase in high-mannan NC diets did not affect jejunal VH:CD ratio in laying hens.

Previous meta-analysis demonstrated that dietary β-mannanase supplementation increased VH, but decreased CD, thereby increasing VH:CD ratio in the small intestine of broiler chickens [21,24]. This positive effect of intestinal morphology has been involved in decreased digesta viscosity by dietary β-mannanase [21,25]. Digesta viscosity is highly related to intestinal cell development because increasing digesta viscosity facilitates the turnover of intestinal cells [25]. Moreover, decreased β-mannan contents in the GIT by dietary β-mannanase may decrease immune stimulations in the intestinal tissues, leading to an increase in energy and nutrient supply for intestinal cells [22]. It has also been reported that increasing supplementation of dietary β-mannanase linearly increased VH:CD ratio in broiler chickens [21,26]. However, in contrast to these previous findings, dietary β-mannanase supplementation in high-mannan NC diets did not affect jejunal VH, CD, and VH:CD ratio in the present study. Interestingly, laying hens fed high-mannan NC diets, regardless of dietary β-mannanase supplementation, had decreased VH:CD ratio than those fed PC or NC diets. The reason for this observation may be associated with high concentrations of fiber in PKM and CM because high amounts of fiber in diets have been frequently associated with impaired intestinal morphology and development in poultry [27,28].

### Blood measurements

All blood measurements, including H:L ratio, AST, ALT, uric acid, and creatinine, were not affected by dietary treatments (Table 6). The serum concentrations of AST and ALT as biomarkers of liver health and functions [29], and uric acid and creatinine as biomarkers of kidney health and functions [30] have been widely determined in poultry studies. However, no effects of dietary treatments on their serum concentrations were identified in this study, which may indicate that reduction in dietary energy and protein levels used in this study, the inclusion of 2.5% PKM and 2.5% CM, and dietary β-mannanase supplementation exert no adverse effects on health and functions of liver and kidney in laying hens. It appears that laying hens used in this study were in an early and peaking stage, and therefore, they may display a high health status of liver and kidney, thereby explaining little impacts of dietary treatments on blood measurements in the current study.

The blood H:L ratio has been accessed as a stress indicator in poultry, with its increasing values representing increased stress levels in poultry [31]. However, this study revealed that dietary treatments did not affect blood H:L ratio in laying hens. This result may indicate that the current experimental diets with reduced protein and energy levels, inclusion of high-mannan ingredients, and dietary β-mannanase supplementation do not largely induce stress responses in laying hens. It has been reported that nutritional deficiency may lead to a stress response in poultry, possibly due to compromised gut-brain axis [32,33]. Based on this previous result, it was expected that low-energy and low-protein diets used in this study may also induce stress responses; however, such an effect was not found in this study. Therefore, it can be inferred that the moderate reduction in energy and protein levels used in this study does not promote stress levels in laying hens.

### Immune response

**Figure 1:**
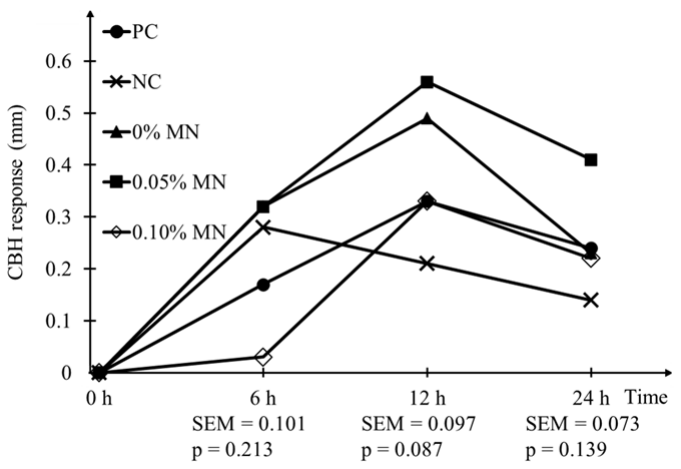
Effect of dietary β-mannanase (MN) supplementation in low-energy and low-protein diets containing high-mannan ingredients on cutaneous basophil hypersensitivity (CBH) response in laying hens. Data are least squares means of 8 observations per treatment. PC, corn-soybean meal-based diet with 2,950 kcal/kg AME_n_ and 17.0% CP; NC, corn-soybean meal-based diets with low-energy and low-protein diets with 2,850 kcal/kg AME_n_ and 16.15% CP; 0% MN, high-mannan NC diets with no MN supplementation; 0.05% MN, high-mannan NC diets with 0.05% MN supplementation; 0.10% MN, high-mannan NC diets with 0.10% MN supplementation; SEM, standard error of means at each time.

A tendency (p = 0.087) for the least values for 12-h CBH tests was observed in laying hens fed NC diets among dietary treatments (Figure 1). However, dietary treatments did not influence the values for 6-h and 24-h CBH tests.

The CBH test has been used to measure cell-mediated immune responses in poultry with increasing CBH values reflecting a potential improvement in immune responses [34]. Although the significance was not detected, dietary β-mannanase supplementation, in particular at the level of 0.05% appeared to increase CBH values compared with NC treatment. There have been several studies reporting that dietary β-mannanase improves immune responses in poultry [22]. The reason has been related to the prebiotic effect of mannan-oligosaccharides derived from the breakdown of β-mannan, following dietary β-mannanase supplementation [3]. However, the positive effect of β-mannanase was considerable only at the supplemental level of 0.05%, with no effects at the supplemental level of 0.10%. The reason for this observation remains unknown because limited information regarding the effect of dietary β-mannanase on immune responses in poultry has been available.

### Fatty liver incidence

Hens fed NC diets had a greater (p<0.05) subjective color score in the liver than those fed PC diets or high-mannan NC diets with 0.05% or 0.10% β-mannanase supplementation (Table 7). Likewise, feeding NC diets showed a greater (p<0.05) hemorrhagic score in the liver than feeding other treatment diets. There was a tendency (p = 0.086) for the highest L* value in hens fed NC diets, but other CIE color values (a* and b*) did not differ among dietary treatments. The concentrations of AEE in the liver were also unaffected by dietary treatments.

Fatty liver and fatty liver hemorrhagic syndrome are commonly observed in laying hens because of increasing fat retention as hens are aged [12,35]. In this study, feeding NC diets showed the highest incidence of fatty liver problems as observed in subjective color score and hemorrhagic score. This observation is likely contradictory to the general knowledge that high energy intake increases fat synthesis, whereas low energy intake decreases fat synthesis in the body [36,37]. However, our previous study has suggested that this knowledge is not always true because energy sources may have a greater impact on fat synthesis rather than total energy intake [12,35]. The NC diets in this study contained the least amounts of fat to decrease energy concentrations in the treatment diet. Decreasing fat concentrations in NC diets may even facilitate hepatic fat synthesis as reported in our previous study [12,35], which may be the reason why NC diets increased fatty liver problems with the highest liver color scores in this study. However, analyzed fat concentrations in the liver were not largely affected by dietary treatments. On the contrary, feeding high-mannan NC diets resulted in decreased subjective color and hemorrhagic scores in the liver compared with NC diets although energy and protein concentrations were equalized between NC and high-mannan NC diets. The reason may also be associated with high concentrations of fiber in high-mannan diets because feeding high-fiber diets decreased fatty liver incidence by decreasing hepatic fat retention [12,38]. However, dietary supplementation of β-mannanase in high-mannan NC diets had no positive effect on fatty liver incidence in this study.

## CONCLUSION

Most of productive performance and egg quality in laying hens were not affected by reduction of 100 kcal/kg AME_n_ and 0.85% protein in diets, inclusion of high-mannan ingredients (2.5% PKM and 2.5% CM), and dietary supplementation of 0.05% and 0.10% β-mannanase in high-mannan diets. However, inclusion of high-mannan ingredients in low-energy and low-protein diets decreased VH:CD ratio and fatty liver incidence in laying hens. Finally, 0.05% and 0.10% β-mannanase supplementation in low-energy and low-protein diets containing high-mannan ingredients had little impacts on productive performance, egg quality, intestinal morphology, and liver health in laying hens. Further studies may be warranted to investigate the effect of dietary β-mannanase supplementation in layer diets that include varying levels of high-mannan ingredients, as well as different reducing extents of energy and protein levels.

## Figures and Tables

**Table 1:** Analyzed nutrient concentrations of the palm kernel meal and copra meal used in this experiment (%, as-fed basis)

Items	Palm kernel meal	Copra meal
Dry matter (%)	90.8	89.1
Gross energy (kcal/kg)	4,317	3,946
Crude protein (%)	16.20	21.26
Neutral detergent fiber (%)	53.84	48.97
Acid detergent fiber (%)	30.30	23.03
Ether extract (%)	5.77	3.00
Amino acids (%)		
Trp	0.14	0.15
Arg	1.54	1.50
His	0.28	0.31
Ile	0.50	0.53
Leu	1.04	1.16
Lys	0.48	0.35
Met	0.39	0.33
Phe	0.63	0.74
Thr	0.49	0.56
Val	0.74	0.82
Ala	0.67	0.80
Asp	1.16	1.37
Cys	0.31	0.42
Glu	2.72	3.26
Gly	0.70	0.77
Pro	0.60	0.73
Ser	0.61	0.75
Tyr	0.29	0.32

**Table 2:** Composition and nutrient concentrations of experimental diets

Items	PC	NC	High-mannan NC
Ingredients (%)			
Corn	60.650	60.050	59.640
Soybean meal (45% CP)	10.120	18.250	7.650
Corn gluten meal	11.100	4.770	9.780
Palm kernel meal	0.000	0.000	2.500
Copra meal	0.000	0.000	2.500
Soybean oil	3.976	3.550	3.460
Monodicalcium phosphate	1.971	1.940	1.965
Limestone	9.137	9.096	9.134
l-Lysine HCl	0.418	0.235	0.488
dl-Methionine	0.183	0.237	0.223
l-Threonine	0.081	0.072	0.124
l-Tryptophan	0.067	0.039	0.079
l-Arginine	0.137	0.000	0.154
l-Isoleucine	0.061	0.056	0.115
l-Valine	0.049	0.055	0.098
Celite	0.200	0.200	0.200
Salt	0.200	0.200	0.200
50% Choline	0.250	0.250	0.250
NaHCO_3_	0.600	0.340	0.550
K_2_CO_3_	0.400	0.260	0.490
Coccidiostat	0.050	0.050	0.050
Antioxidant	0.050	0.050	0.050
Vitamin premix^1)^	0.150	0.150	0.150
Mineral premix^2)^	0.150	0.150	0.150
Total	100.000	100.000	100.000
Energy and nutrient contents^3)^			
AME_n_ (kcal/kg)	2,950	2,850	2,850
CP (%)	17.00	16.15	16.15
Digestible Lys (%)	0.79	0.79	0.79
Digestible Met (%)	0.47	0.49	0.49
Digestible Met+Cys (%)	0.71	0.71	0.71
Digestible Thr (%)	0.55	0.55	0.55
Digestible Trp (%)	0.17	0.17	0.17
Digestible Arg (%)	0.82	0.82	0.82
Digestible Ile (%)	0.63	0.63	0.63
Digestible Val (%)	0.70	0.70	0.70
Total calcium (%)	3.81	3.81	3.81
Available phosphorus (%)	0.41	0.41	0.41

PC, corn-soybean meal-based diet with recommended energy and CP levels; NC, corn-soybean meal-based diets with low-energy and low-protein diets (decreased AME_n_ by 100 kcal/kg and CP by 0.85% than PC diets); High-mannan NC, low-energy and low-protein NC diets formulated with 2.5% palm kernel meal and 2.5% copra meal.

1) Provided per kg of the complete diet: vitamin A, 12,000 IU (retinyl acetate); vitamin D_3_, 4,000 IU; vitamin E, 80.0 mg; vitamin K_3_, 4.0 mg (menadione dimethylpyrimidinol); vitamin B_1_, 4.0 mg; vitamin B_2_, 10.0 mg; vitamin B_6_, 6.0 mg; vitamin B_12_, 20.0 μg; folic acid, 2.0 mg; biotin, 200 μg; niacin, 60 mg.

2) Provided per kg of the complete diet: iron, 60 mg (FeSO_4_); zinc, 100 mg (ZnSO_4_); manganese, 120 mg (MnO); copper, 16 mg (CuSO_4_); cobalt, 1,000 μg (CoSO_4_); selenium, 300 μg (Na_2_SeO_3_); iodine, 1.25 mg [Ca(IO_3_)_2_].

3) Calculated values from CVB [39].

**Table 3:** Effect of dietary β-mannanase (MN) supplementation in low-energy and low-protein diets containing high-mannan ingredients on productive performance in laying hens

Item	Dietary treatments^1)^					SEM	p-value

	PC	NC	High-mannan NC				

			0% MN	0.05% MN	0.10% MN		
HD (%)	95.8	95.7	96.0	95.2	94.7	0.69	0.642
EW (g)	60.8	61.8	60.9	61.2	61.1	0.36	0.360
EM (g)	58.3	59.2	58.5	58.3	57.8	0.55	0.560
FI (g/hen/d)	103^c^	105^ab^	106^a^	105^ab^	104^bc^	0.75	0.038
FCR (g/g)	1.77	1.78	1.82	1.80	1.80	0.018	0.339
BS (%)	0.52	0.84	0.36	0.35	0.43	0.165	0.222

Data are least squares means of 8 observations per treatment.

1) PC, corn-soybean meal-based diet with 2,950 kcal/kg AME_n_ and 17.0% CP; NC, corn-soybean meal-based diets with low-energy and low-protein diets with 2,850 kcal/kg AME_n_ and 16.15% CP; High-mannan NC, low-energy and low-protein NC diets formulated with 2.5% palm kernel meal and 2.5% copra meal.

a–c Means with different superscripts within a row differ (p<0.05).

HD, hen-day egg production; EW, egg weight; EM, egg mass; FI, feed intake; FCR, feed conversion ratio; BS, broken and shell-less egg production rate.

**Table 4:** Effect of dietary β-mannanase (MN) supplementation in low-energy and low-protein diets containing high-mannan ingredients on egg quality in laying hens

Item		Dietary treatments^1)^						

		PC	NC	High-mannan NC			SEM	p-value

				0% MN	0.05% MN	0.10% MN		
Eggshell color (shell color fan)		12.1	11.8	11.9	12.5	12.0	0.24	0.263
Eggshell color (CIE Lab value)	L*	49.7	50.1	49.8	48.8	49.6	0.35	0.122
	a*	20.5	20.3	20.6	20.9	20.6	0.20	0.282
	b*	29.2	29.3	29.4	29.3	29.5	0.23	0.950
Egg yolk color (Roche color fan)		8.8^a^	7.3^c^	8.8^a^	8.8^a^	7.9^b^	0.10	<0.001
Eggshell strength (kg/cm^2^)		4.32	4.42	4.56	4.57	4.36	0.153	0.686
Eggshell thickness (μm)		362	367	366	368	364	5.4	0.942
Haugh unit		97.3	97.5	98.8	97.1	98.0	1.60	0.947

Data are least squares means of 8 observations per treatment.

1) PC, corn-soybean meal-based diet with 2,950 kcal/kg AME_n_ and 17.0% CP; NC, corn-soybean meal-based diets with low-energy and low-protein diets with 2,850 kcal/kg AME_n_ and 16.15% CP; High-mannan NC, low-energy and low-protein NC diets formulated with 2.5% palm kernel meal and 2.5% copra meal.

a–c Means with different superscripts within a row differ (p<0.05).

CIE, Commission Internationale de l′Eclairage; L*, Lightness; a*, redness; b*, yellowness.

**Table 5:** Effect of dietary β-mannanase (MN) supplementation in low-energy and low-protein diets containing high-mannan ingredients on jejunal morphology in laying hens

Item	Dietary treatments^1)^					SEM	p-value

	PC	NC	High-mannan NC				

			0% MN	0.05% MN	0.10% MN		
VH (μm)	1,051	1,096	1,004	1,056	1,088	66.9	0.879
CD (μm)	190	198	210	217	224	14.1	0.441
VH:CD ratio	5.95^a^	5.90^a^	4.79^b^	4.97^b^	4.86^b^	0.256	0.002

Data are least squares means of 8 observations per treatment.

1) PC, corn-soybean meal-based diet with 2,950 kcal/kg AME_n_ and 17.0% CP; NC, corn-soybean meal-based diets with low-energy and low-protein diets with 2,850 kcal/kg AME_n_ and 16.15% CP; High-mannan NC, low-energy and low-protein NC diets formulated with 2.5% palm kernel meal and 2.5% copra meal.

a,b Means with different superscripts within a row differ (p<0.05).

VH, villus height; CD, crypt depth.

**Table 6:** Effect of dietary β-mannanase (MN) supplementation in low-energy and low-protein diets containing high-mannan ingredients on blood measurements in laying hens

Item	Dietary treatments^1)^					SEM	p-value

	PC	NC	High-mannan NC				

			0% MN	0.05% MN	0.10% MN		
Blood H:L ratio	0.22	0.23	0.25	0.22	0.24	0.026	0.924
Serum levels							
AST (U/L)	151.2	151.5	106.2	149.1	146.1	16.92	0.279
ALT (U/L)	1.44	1.20	0.86	0.98	0.84	0.229	0.419
Uric acid (mg/dL)	5.24	3.60	2.75	3.57	4.38	0.810	0.271
Creatinine (mg/dL)	0.19	0.18	0.14	0.19	0.19	0.020	0.371

Data are least squares means of 8 observations per treatment.

1) PC, corn-soybean meal-based diet with 2,950 kcal/kg AME_n_ and 17.0% CP; NC, corn-soybean meal-based diets with low-energy and low-protein diets with 2,850 kcal/kg AME_n_ and 16.15% CP; High-mannan NC, low-energy and low-protein NC diets formulated with 2.5% palm kernel meal and 2.5% copra meal.

H:L ratio, heterophil to lymphocyte ratio; AST, aspartate aminotransferase; ALT, alanine aminotransferase.

**Table 7:** Effect of dietary β-mannanase (MN) supplementation in low-energy and low-protein diets containing high-mannan ingredients on fatty liver incidence in laying hens

Item		Dietary treatments^1)^					SEM	p-value

		PC	NC	High-mannan NC				

				0% MN	0.05% MN	0.10% MN		
Subjective color score		1.33^bc^	2.38^a^	1.88^ab^	1.25^c^	1.58^bc^	0.213	0.004
CIE color value	L*	27.8	31.1	30.5	27.1	29.4	1.15	0.086
	a*	18.9	19.0	18.8	18.3	20.2	1.06	0.802
	b*	9.7	12.2	11.8	9.6	11.1	0.96	0.213
Hemorrhagic score		0.21^b^	1.29^a^	0.63^b^	0.17^b^	0.46^b^	0.220	0.006
AEE (% DM)		24.6	20.3	24.3	19.1	21.1	1.78	0.139

Data are least squares means of 8 observations per treatment.

1) PC, corn-soybean meal-based diet with 2,950 kcal/kg AME_n_ and 17.0% CP; NC, corn-soybean meal-based diets with low-energy and low-protein diets with 2,850 kcal/kg AME_n_ and 16.15% CP; High-mannan NC, low-energy and low-protein NC diets formulated with 2.5% palm kernel meal and 2.5% copra meal.

a–c Means with different superscripts within a row differ (p<0.05).

CIE, Commission Internationale de l′Eclairage; L*, Lightness; a*, redness; b*, yellowness; AEE, acid-hydrolyzed ether extract.
